# 

**Published:** 1878-06

**Authors:** 


					﻿Books and Pamphlets Received.
Vest Pocket Anatomist, by C. Henri Leonard, A. M., M. D.
Congenital Occlusion and Dilatation of Lymph Channels, by Samuel C.
Bussey, M. D. New York : Wm. Wood & Co. 1878. Buffalo: H. H. Otis.
Thirty-fifth Annual Report of the Managers of the State Lunatic Asylum,
Utica, N. Y., for the year 1877.
President’s Address before the Medical Society of West Virginia, by E. A.
Hildreth, M. D. Wheeling. 1877.
Annual Announcement of the Medical Department of the University of
California, Session of 1878.
Observations in Practice, Surgery, Gynecology, and especially Obstetrics,
by George B. Walker, M. D. Reprinted from the Chicago Medical Journal
and Examiner, February and March, 1878.
Catalogue of Lafayette College, 1877-78.
Popular Science Monthly, May and June, 1878, and Supplement, April and
May.
Dangers from Color-Blindness in Railroad Employes and Pilots, by B. Joy
Jeffries, A. M., M. D. (Harv.), Boston. 1878.
THE BUFFALO
Medical and Surgical Journal
FTTBLISHED ZbZlOI^TUZLrTV
Containing Original Articles, Reports of Medical Societies and Hospitals, Edito-
rial, Reviews, Correspondence, Army News, etc., etc ; including the usual variety
of Medical Periodical Publications. Specimen copies sent on application.
Address Buffalo Medical & Surgical Journal, Buffalo, N. Y.
TERMS OF SUBSCRIPTION, $3.00 PER YEAR, IN ADVANCE.
All communications for publication shoud be sent before the fifteenth (15th) of each
month, or they will of necessity lie over until the next number. No change can be made in
advertisements after the twenty-fifth. Address, Buffalo Medical and Surgical Journal, 978
Main Street.
Correspondence and original articles are solicited. Publication is no evidence that the
views of the author are endorsed.
				

